# The Service of Research Analytics to Optimize Digital Health Evidence Generation: Multilevel Case Study

**DOI:** 10.2196/14849

**Published:** 2019-11-11

**Authors:** Quynh Pham, James Shaw, Plinio P Morita, Emily Seto, Jennifer N Stinson, Joseph A Cafazzo

**Affiliations:** 1 Institute of Health Policy, Management, and Evaluation Dalla Lana School of Public Health University of Toronto Toronto, ON Canada; 2 Centre for Global eHealth Innovation Techna Institute University Health Network Toronto, ON Canada; 3 Women's College Hospital Institute for Health System Solutions and Virtual Care Toronto, ON Canada; 4 School of Public Health and Health Systems Faculty of Applied Health Sciences University of Waterloo Waterloo, ON Canada; 5 Department of Anesthesia and Pain Medicine The Hospital for Sick Children Toronto, ON Canada; 6 Lawrence S Bloomberg Faculty of Nursing University of Toronto Toronto, ON Canada; 7 Child Health Evaluative Sciences Research Institute The Hospital for Sick Children Toronto, ON Canada; 8 Institute of Biomaterials and Biomedical Engineering Faculty of Applied Science and Engineering University of Toronto Toronto, ON Canada

**Keywords:** research analytics, effective engagement, digital health, mobile health, implementation, log data, service design, chronic disease

## Abstract

**Background:**

The widespread adoption of digital health interventions for chronic disease self-management has catalyzed a paradigm shift in the selection of methodologies used to evidence them. Recently, the application of digital health research analytics has emerged as an efficient approach to evaluate these data-rich interventions. However, there is a growing mismatch between the promising evidence base emerging from analytics mediated trials and the complexity of introducing these novel research methods into evaluative practice.

**Objective:**

This study aimed to generate transferable insights into the process of implementing research analytics to evaluate digital health interventions. We sought to answer the following two research questions: (1) how should the service of research analytics be designed to optimize digital health evidence generation? and (2) what are the challenges and opportunities to scale, spread, and sustain this service in evaluative practice?

**Methods:**

We conducted a qualitative multilevel embedded single case study of implementing research analytics in evaluative practice that comprised a review of the policy and regulatory climate in Ontario (macro level), a field study of introducing a digital health analytics platform into evaluative practice (meso level), and interviews with digital health innovators on their perceptions of analytics and evaluation (microlevel).

**Results:**

The practice of research analytics is an efficient and effective means of supporting digital health evidence generation. The introduction of a research analytics platform to evaluate effective engagement with digital health interventions into a busy research lab was ultimately accepted by research staff, became routinized in their evaluative practice, and optimized their existing mechanisms of log data analysis and interpretation. The capacity for research analytics to optimize digital health evaluations is highest when there is (1) a collaborative working relationship between research client and analytics service provider, (2) a data-driven research agenda, (3) a robust data infrastructure with clear documentation of analytic tags, (4) in-house software development expertise, and (5) a collective tolerance for methodological change.

**Conclusions:**

Scientific methods and practices that can facilitate the agile trials needed to iterate and improve digital health interventions warrant continued implementation. The service of research analytics may help to accelerate the pace of digital health evidence generation and build a data-rich research infrastructure that enables continuous learning and evaluation.

## Introduction

### Background

The widespread adoption of digital health interventions for chronic disease self-management has catalyzed a paradigm shift in the selection of methodologies used to evidence them [1]. This turn toward alternative research designs and methods is predicated on the understanding that traditional approaches to evidence generation cannot keep pace with digital health innovation [2]. Transformative advances to the technological components powering these novel interventions have rapidly changed their capacity to improve health outcomes [3]. As such, they demand agile and iterative evaluations that can continuously measure their effects in real time [4]. To address this methodological challenge and generate timely insights, digital health scholars have operationalized research protocols that capitalize on the unique characteristics of these technology mediated interventions [5-7]. Scholarship on optimizing digital health trials has ranged from leveraging smartphone ubiquity to accelerate research recruitment and informed consent [8], to harnessing sensor capabilities to capture novel clinical endpoints [8], to employing research designs and methods from the engineering sciences that can rapidly yield actionable outcomes [9].

Recently, the application of *digital health analytics*, defined as “the discovery and communication of patterns in health data,” has emerged as an efficient approach to evaluate digital health interventions [10]. Numerous evaluative endeavors have mined the rich log data generated by users engaging with these inventions and successfully generated evidence of their impact on health outcomes [11]. In turn, the analytic models derived from these efforts have enhanced intervention effects through identifying the mediating behavioral mechanisms that motivate improved outcomes [12-14]. Scholars have also pushed for new theoretical frameworks to guide more systematic log data analyses and support transparent and replicable evidence generation [15]. The aggregation of this research productivity has advanced the scale and spread of research analytics in industry and academic evaluative practice.

In March 2018, our research group sought to contribute a resource to support applying analytic research methods to evaluate digital health interventions. The Analytics Platform to Evaluate Effective Engagement (APEEE) with digital health interventions was developed to facilitate the quantification, analysis, and visualization of research data [16]. The platform provides investigators with the means to characterize the breadth and depth of digital health engagement required to change behaviors and achieve intended health outcomes. With APEEE, investigators are able to cull through large, dense, and dynamic datasets in real time and identify meaningful patterns of digitally mediated behavior change. They can apply this functionality to conduct analytic evaluations and generate timely evidence to optimize intervention effectiveness. A formative evaluation of APEEE showed that digital health researchers perceived the platform to be an acceptable evaluative resource and were satisfied with its design, functionality, and performance [16]. They saw potential in APEEE to accelerate and augment evidence generation and expressed enthusiasm for adopting the platform to support their evaluative practice once fully implemented. However, more implementation research was required to formally evaluate the impact of the platform on digital health evaluative practice.

Although analytic methodologies have shown promise as an alternative approach to evidencing digital health interventions, they have not been without limitations. Trials have been small, research processes are often ad hoc and reactive, and barriers to introducing and sustaining new practices are rarely discussed [17]. Traditional research operations may be slow and cumbersome, but they benefit from a legacy of checks and balances that have been honed to ensure the valid collection of outcome measures [2]. There is currently no equivalent to these standard operating procedures to address analytic issues of missing or erroneous trial data, owing to digital device failure or participant disengagement from these devices [18]. Concerns regarding privacy and data security in digital health care delivery have also extended to digital health research [19]. Aggregating data from multiple sources for the purpose of applying research analytics will require the adoption of standards, raise privacy and ethical concerns, and demand new ways to preserve privacy [20]. Significant time, effort, and resources are required to develop the capacity to conduct data-driven research, particularly to set up the technological infrastructure, and also to train and support both staff and patients in this new practice.

### Objectives

In short, there is a growing mismatch between the promising evidence base emerging from digital health analytics and the complexity of introducing these novel research methods into evaluative practice. Limited research exists on implementation challenges, notably the policy and regulatory climate required to support new approaches to digital health evidence generation, the practicalities of organizational change to accommodate analytic methodologies, and the personal narratives of digital health innovators who engage in these practices. To address these knowledge gaps, we sought to explore a new implementation of research analytics in a digital health research lab at the Hospital for Sick Children (SickKids) in Toronto, Canada, and draw wider insights on the contextual factors that enable and constrain analytic models of evaluation.

## Methods

### Research Overview

This study aimed to generate transferable insights into the process of implementing research analytics to evaluate digital health interventions.

We sought to answer the following two research questions: (1) how should the service of research analytics be designed to optimize digital health evidence generation and (2) what are the challenges and opportunities to scale, spread, and sustain this service in evaluative practice?

Our objectives were as follows:

1. At the macro level, to review policy and regulatory mandates for evidencing digital health interventions and assess whether research analytics can generate acceptable levels of evidence.
2. At the meso level, to explore the sociotechnical systems and services that enable operationalizing research analytics into evaluative practice.
3. At the micro level, to understand the personal motivations of digital health innovators for applying research analytics to evidence their products.

### Study Design

We conducted a qualitative multilevel embedded single case study of implementing digital health research analytics in evaluative practice that comprised a review of the policy and regulatory climate in Ontario (macro level), a field study of APEEE’s introduction into evaluative practice (meso level), and interviews with digital health innovators on their perceptions of analytics and evaluation (micro level). Employing an embedded single case study design allowed us to coalesce multiple levels of mutually shaping contexts that influence a representative case of digital health evidence generation [21]. All embedded units of analysis are summarized in Table 1 and described below.

**Table 1:** Overview of embedded units of analysis.

Embedded units of analysis	Type and nature of data
Macro-level study of the provincial context	Provincial policy and regulatory documents; provincial stakeholder working group field notes and meeting minutes; informal unstructured interviews with 5 senior leaders
Meso-level study of organizational change	Accounts of 5 research staff involved in conducting digital health evaluations; approximately 20 hours of observations in a digital health research lab; implementation artifacts
Microlevel study of personal motivations	Formal semistructured interviews with 21 digital health innovators

### Data Collection

#### Macro Level

A broad literature review of recent provincial documents (eg, policies, frameworks, and funding programs) was conducted to assess the current digital health context. We sought to discern strategic interests for evidencing digital health interventions, with a specific interest in identifying barriers and facilitators to implementing analytic research methods. In parallel, between January 2017 and March 2019, we attended 7 working group sessions on sustaining and evaluating digital health innovations hosted as part of *Project SPARK*, a collaborative partnership between the Ministry of Health and Long-Term Care (MOHLTC), eHealth Ontario, the MaRS Discovery District, and the University Health Network in Toronto, Canada [22,23]. This partnership was formed to bring together thought leaders who shared a common vision to stimulate consumer digital health innovation in Ontario. Approximately 10 to 15 stakeholders across partnership organizations were present at each meeting. Topics discussed included the challenge of sustaining and evaluating digital health applications that were connected to provincial health assets (eg, Ontario Laboratories Information System and Digital Health Drug Repository). Field notes and meeting minutes were retained for analysis. Finally, we conducted informal unstructured interviews with a convenience sample of 5 senior leaders from academic (n=3) and industry (n=2) organizations to seek corroboration and clarification on the policy and regulatory directives identified in our literature review. These interviews were not audiotaped in accordance with informant preferences, but we obtained verbal consent to record interview notes for analysis.

#### Meso Level

To gain a real-world understanding of how to introduce digital health research analytics into evaluative practice, we conducted a 6-month field study of APEEE’S inaugural implementation in the Improving Outcomes in Child Health through Technology (iOUCH) lab at SickKids. The iOUCH lab aims to improve the lives of children and adolescents through the use of innovative information and communication technologies [24]. The research group comprises a principal investigator, a PhD-prepared associate, numerous managers, coordinators, and analysts, and a rotating roster of students and fellows, for a total of over 20 research staff. The group conducts research to conceptualize, design, and evidence digital health interventions and outsources the development of the interventions to external research groups or software development studios. This field study follows the preliminary work conducted by our research group to design and develop APEEE and connect it to *iCanCope*, a smartphone-based pain self-management program tailored for young people aged 12 to 25 years with chronic pain [25]. The app prompts them to check in every day and report their symptoms, set goals to improve their pain and function, read about pain coping strategies, and get social support from other young people living with chronic pain. *iCanCope* was conceived by the iOUCH lab, with our research group as the development partner [26]. The app also served as a use case to inform APEEE’s product specifications during the design and development of the platform [16]. This existing partnership and history of research collaboration provided a primed setting for us to study the reality of setting up and delivering the service of research analytics in a digital health research lab.

We aimed to map the network of people, tasks, and organizational routines that were required to support APEEE and the changes in these interactions and interdependencies over time. This sociotechnical approach was operationalized through conducting 5 half-day observation sessions with 5 research staff (ie, 1 associate, 1 manager, 1 coordinator, and 2 analysts) who worked on the *iCanCope* project. We observed staff engaging in their daily routines throughout the course of the study to capture changes in evaluative practice. Field notes were recorded during each observation session for analysis. We supplemented these observation sessions through conducting 30-min interviews with all 5 staff members at the start and end of the field study. A semistructured interview script was used to elicit expectations for how APEEE might change practice and, consequently, whether these changes occurred as expected. In addition to the abovementioned research activities, we were in a unique position to be responsible for supporting the introduction of APEEE into the iOUCH lab and the subsequent maintenance of the service. In our role as the APEEE product team, we conducted training sessions, made ad hoc changes and updates to the platform, drafted guidance documentation, and provided ongoing client support. This environment allowed us to experience a vendor-client relationship and react to real implementation challenges. Through this study, we were able to study the process of implementing APEEE in ethnographic detail and catalog the services and artifacts that were produced, both planned and improvised.

#### Micro Level

We approached 33 digital health innovators to participate in a 30- to 45-min audiotaped semistructured interview on their perceptions of digital health evaluation and analytics. A purposive sampling method was employed where innovators who were known to the research team were invited to participate in this research. We also sought maximum variation in innovator sector, occupation, and rank to capture a broad range of contexts and motivations.

The following criteria were applied to purposefully select digital health innovators for inclusion in this study:

1. Adults aged 18 years or older
2. Conversant in English
3. Identified by the research team as a digital health innovator (we define a digital health innovator as both academic and industry professionals who are involved in the design, development, and evaluation of digital health interventions; this includes scientists, clinicians, project managers, research coordinators, chief executive officers, chief technical officers, statisticians, developers, designers, data architects, and other relevant positions)
4. Previous or current involvement on a digital health project
5. Interest in evaluating the impact of a digital health intervention

All identified innovators were first contacted through a study recruitment email sent by the lead researcher. This email contained a brief description of the study objectives as well as a succinct summary of study activities and considerations (eg, data privacy and confidential quotes from any transcripts). If innovators expressed interest in joining the study, they were scheduled for an interview. Consent was obtained verbally and audiotaped before the start of the interview. Participants were not compensated for their involvement in this research. Ethics approval for this study was obtained from the University of Toronto Research Ethics Board (reference number 00035682).

### Data Analysis

#### Analytical Framework

Our analysis drew from Greenhalgh et al’s diffusion of innovations model to characterize the complexity of innovating digital health evaluative practice [27]. This theoretical model has been widely used to study the scale and spread of health innovations into organizational practice. We were motivated by Greenhalgh et al’s own use of this model in a case study exploring the introduction of shared electronic patient records (EPR) into care sites in the United Kingdom [28]. Although EPRs may be specific to health care settings, the data sharing mechanisms that power them are context-agnostic. The access that EPRs provide to clinical data networks is comparable with the access that analytic resources provide to research data repositories. As such, the diffusion of innovations model allowed us to explain and interpret (1) the complexity of implementing digital health research analytics in evaluative practice; (2) the dynamic nature of the implementation process; and (3) the shifting political, organizational, and technological context over time. We also heavily referenced Greenhalgh et al’s work on the real-world implementation of video outpatient consultations in UK clinical practices to structure our research according to macro, meso, and micro levels of analysis and produce a cohesive story of how the digital health research system reacts to methodological change [29].

#### Process

Our efforts to map the sociotechnical digital health research system provided detailed data on the challenges of operationalizing research analytics and the workarounds to overcome them. We amassed data on issues related to the technology, the research environment, administrative processes, and data privacy and governance. This research generated various types of qualitative data (ie, documents, field notes, and interview transcripts), which we analyzed separately using the directed content analysis approach [30]. With Greenhalgh et al’s diffusion of innovations model as our conceptual anchor, we identified key components from the model to serve as initial coding categories [27]. We developed operational definitions for all relevant components of the model, assigned a code to each component, and then sorted through our data to identify themes and phrases that could be organized under our theory-informed codes. We used first-order codes to categorize our data (eg, *attributes of the technology as an innovation* and *organizational antecedents for innovation*), with second-order codes fleshing these out in greater detail (eg, under *organizational antecedents for innovation* were subcategories of *relative advantage* and *organizational slack*, among others). Once coding was completed, we thematically analyzed within and across the three embedded units of analysis to detail the reconfiguration of organizational routines that accompanied the introduction of research analytics. This thematic investigation allowed us to surface the tensions between current *research as usual* and new ways of conducting digital health research facilitated by analytics. Our findings are reported in a narrative case format [31,32], with direct quotes and attributions embedded into the text (eg, P1) to convey the style and intent through which participants expressed their thoughts and experiences [33].

## Results

### Macro-Level Findings

Currently, there is a strong policy push in Ontario to build digital health capacity. This agenda was initiated in February 2015 by the MOHLTC with the launch of their *Patients First: Action Plan for Health Care* [34]. Designed to “put people and patients first by improving their health care experience and their health outcomes,” the plan outlined four key objectives to place the patient at the center of the health care system and shift care from hospital to home: (1) provide faster access to care, (2) deliver connected care in the community, (3) keep patients informed to facilitate their health decisions, and (4) protect the public health care system through sustainable policy practices. In support of this proposed health system transformation, the *Patients First: Digital Health Strategy* was published in November 2016 to “advance modern, integrated, patient-centred care” [35]. During his keynote presentation at the 2016 Canada Health Infoway Partnership Conference on the strategy, Deputy Minister Robert Bell reflected that achieving public *Patients First* commitments would require “three building blocks of digital health: strategy, governance, and information management” [35]. This foundation was conjectured to enhance access to health information and services, strengthen health care quality, and stimulate economic growth.

Central to these tenets was the belief that digital health represented positive innovation to the health care system and thus warranted building out infrastructure to modernize existing practices. Of the 7 “guiding principles for digital health” laid out in the digital health strategy, the *Digital First* philosophy was notable in its recommendation that all new and existing programs should be assessed by asking, “how can we do it with digital health?” This enthusiasm was echoed in two major reports commissioned by the Government of Ontario to assess the value of its digital health assets: the Ontario Health Innovation Council and the Advisory Council on Government Assets submitted reports that lauded the potential for the digitalization of Ontario’s health care system to generate “significant and ongoing value and opportunities for patients and families, providers, and the economy” [36,37]. These reports were widely endorsed by industry, academic, and professional organizations across Ontario [38-40]. They consequently led to the creation of numerous strategic digital health funding programs, notably the 4-year Can $20-million Health Technologies Fund to support the early evaluation, procurement, and adoption of innovative technologies into the provincial health care system. In January 2017, the MOHLTC developed the *10-point Digital Health Action Plan* to operationalize its Digital Health Strategy [41]. Both consumer-facing and health system–facing initiatives were identified, and a Digital Health Scorecard was introduced to measure success against quantitative key performance indicators, some of which were ambitious in scope. For example, the scorecard mandated that the number of patients who used a digital health intervention annually should go from 4600 in 2017 to 100,000 in 2021. It was unclear how baseline and projected figures were estimated, what measurement mechanism would capture this growth, or what would happen if performance indicators were not met.

We did not find any provincial policies specifically related to evidencing digital health interventions. The paucity of guidelines or frameworks on acceptable evidence for digital health care technologies made it difficult for us to discern whether analytic research methods were up to standard. Although most of the policy documents we reviewed referred to digital health innovations as being “effective,” “efficient,” “beneficial,” and poised to deliver “value…in areas such as health outcomes, cost avoidance, and jobs” [37], there was no mention of the methods used to evaluate these attributes. However, this hype did not go unquestioned, as concerns were raised by provincial-level stakeholders and documents regarding the effect of health technologies on patient safety and quality of care [36]. Informants noted that there were no systems in place to measure and evaluate the value of these innovations on health outcomes, cost avoidance, and job creation. These apprehensions were primarily assuaged through assurances that “a culture of innovation” led by strong leadership across all levels of the health care system and supported by effective change management would succeed in promoting the adoption of “beneficial health technologies” [42].

Indeed, policy makers saw greater value in brokering partnerships between technology companies and evaluation experts to evidence digital health technologies, in lieu of releasing evidence standards to support building internal evaluation capacity. This belief was operationalized through the launch of two provincial funding programs dedicated to the evaluation of digital health technologies. From 2016 to 2018, 26 digital health technologies were funded a total of Can $10.4 million through the Health Technologies Fund [43]. To qualify for funding, applicants were required to assemble a team comprising a public health service provider organization, a community-based association or advocacy group, a for-profit health technology business, and an established evaluation provider. Teams were not required to use a particular research design or meet a predetermined threshold of evidence. Rather, evaluating a digital health technology served as a strategic means to a specific end goal: the procurement of this technology into the Ontario health care system. In contrast, the province’s decision in October 2017 to allocate Can $1 million in funding for a new Center of Excellence (COE) in Digital Health Benefits Evaluation reflected the renewed value of evidence as a stand-alone policy lever [44,45]. The COE’s mandate was to form a consortium of evaluation partners who would be responsible for conducting the majority of the province’s digital health evaluations and generating “responsive, timely, rapid, robust, high quality evaluations of both innovative and mature digital health assets and digital health technologies” [44]. This call marked the first instance of the province specifying the need for evaluations that could keep pace with the digital health technologies under study. Support for both these initiatives has been mixed; provincial stakeholders endorsed their potential to standardize approaches to digital health evaluation, whereas industry informants expressed reservations that few innovators would manage to secure the partnerships required to qualify for this increasingly exclusive evaluation expertise.

Although provincial policy makers have taken a permissive stance on digital health evidence generation, the federal regulatory bodies that govern these technologies have been more prescriptive. In January 2019, Health Canada released a draft guidance document on the regulation of Software as a Medical Device [46], which sought to classify medical software as meeting the definition of a medical device under the Food and Drugs Act [47], thereby requiring compliance to the Medical Device Regulations [48]. Health Canada also issued a notice of intent to strengthen the postmarket surveillance risk management of class II to IV medical devices [49] and cited the increased availability of real-world data and evidence generated by devices as rationale for this change [50]. The agency intends to propose a series of significant changes to the Medical Device Regulations by Fall 2020, notably providing the Minister of Health with the authority to request (1) *analytical issue reports* from a medical device manufacturer on suspicion of a safety concern and (2) *annual reports* on medical device performance on safety and effectiveness targets [49]. The adoption of analytics as a mechanism to enforce regulatory compliance is significant in its recognition of log data as a valid measure to inform product safety and effectiveness. This enhanced use of real-world evidence throughout the product life cycle was well received by provincial-level stakeholders, who perceive this approach to be aligned with the Digital Health Strategy’s mandate for “increased transparency to guide governance decisions” [35]. Our industry informants were less enthused about potentially having to disclose proprietary information regarding their product’s market performance. They also expressed concerns that failing to meet regulatory targets would have downstream effects on their eligibility for government funding if analytic reports were shared across agencies.

Overall, our analysis identified a small number of policy and regulatory programs with a minor focus on digital health evidence generation, supported through pockets of provincial funding. Provincial-level stakeholders and documents agree in principle that digital health interventions should be supported by a robust body of evidence to warrant public funding. However, the lack of a definitive guidance on what constitutes *good evidence* puts onus on digital health innovators to self-assess the evaluative approach that will yield the greatest return on investment, or to apply for provincial funding schemes that will pair them with an academic evaluation partner. This emerging shift toward a small number of academic groups conducting a large proportion of the province’s digital health evaluations presents an opportunity for the academic sector to drive the provincial digital health research agenda. Although the adoption of research analytics by this sector would rapidly scale this methodology in evaluative practice, the need for sustained funding to maintain the data infrastructure required to operationalize analytic methods is likely to be a significant barrier to provincial rollout.

### Meso-Level Findings

The inaugural implementation of APEEE in the iOUCH lab was characterized by periods of organizational acclimation with intermittent technological iteration. Despite the fact that the research group had been previously engaged in the design and development of the platform and informed its inaugural connection to *iCanCope* [16], implementation proved far more complex and challenging than anticipated. Our approach to introducing the platform into evaluative practice was highly coordinated: we established a small team to manage operations, conducted (1) one 2-hour on-site training session with 3 research staff and (2) two 1-hour video training sessions with 2 research staff to instruct them on APEEE features and functionality, opened a support channel on the Slack collaboration tool to facilitate ongoing communication, and provided staff with guidance documents (eg, product manual, data dictionary, and *frequently asked questions* content) to reference throughout the 6-month trial period. From October 2018 to March 2019, 5 members of the iOUCH research group were able to access APEEE through individual accounts and execute real-time queries across 2.5 years’ worth of *iCanCope* engagement data.

Before APEEE’s introduction into the iOUCH lab, there were signs that the research group was interested in exploring alternative methods of digital health evaluation. Much of the research conducted on *iCanCope* used the randomized controlled trial (RCT) design to generate evidence of clinical efficacy [51]. In their prestudy interviews, research staff disclosed that while they recognized the importance of definitive trials, they also saw value in conducting “quick tests” (P4) to optimize app performance and improve participant engagement. As such, staff were keen to trial novel research methods that would enable them to move away from “business as usual” (P14) and address research questions that their RCTs were not designed to answer. APEEE’s compatibility with these emerging organizational values lowered barriers to adoption but also raised expectations for the platform’s capacity to transform existing practices.

Throughout the course of this study, research staff maintained a perception of APEEE as a “useful” (P1), “reliable” (P2), and “effective” (P13) research resource that “did everything it was supposed to do” (P9). One staff member remarked in their exit interview that the platform “is fantastic but also expected—how could you do this kind of research and not have something like this?” (P9) This positive appraisal of the platform was partly because of its relative advantage in comparison with the analytic process that it ultimately replaced. Before APEEE’s implementation in the iOUCH lab, research staff had to run a command in the Mac operating system Terminal emulator and extract a large comma-separated values file of tabular log data. They were then forced to sift through thousands of log records and parse out the appropriate data fields to resolve their query. With the exception of one staff member who was comfortable building pivot tables in Excel to expedite analyses, all other staff members found the process to be “so overwhelming” (P14) and “unsustainable” (P13) given the increasing volume of participants to be enrolled into trials of *iCanCope*. Overall, staff lamented the “tedious” (P2) and “time-consuming” (P1) effort required to answer “basic questions” (P13), and obtain a “quick snapshot” (P2) of study status and performance. Thus, they saw APEEE as a welcome change to existing processes and stated at their exit interview that they would recommend “anyone doing research on apps like *iCanCope* to have access to APEEE” (P9).

**Figure 1:**
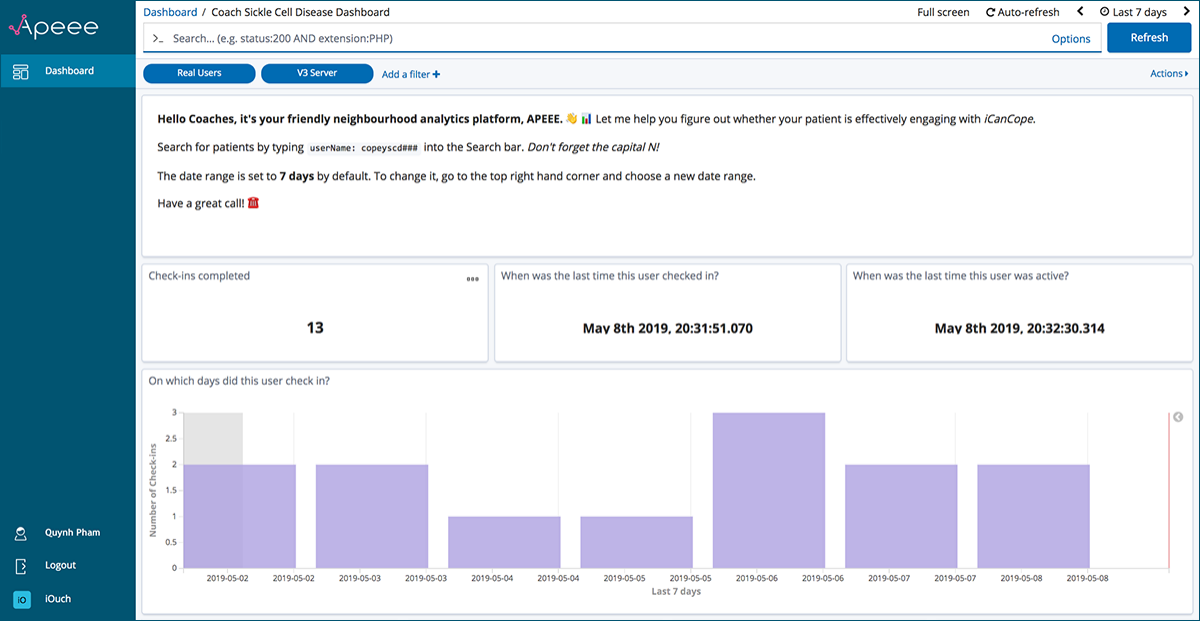
Screenshot of the Analytics Platform to Evaluate Effective Engagement research assistant dashboard.

**Figure 2:**
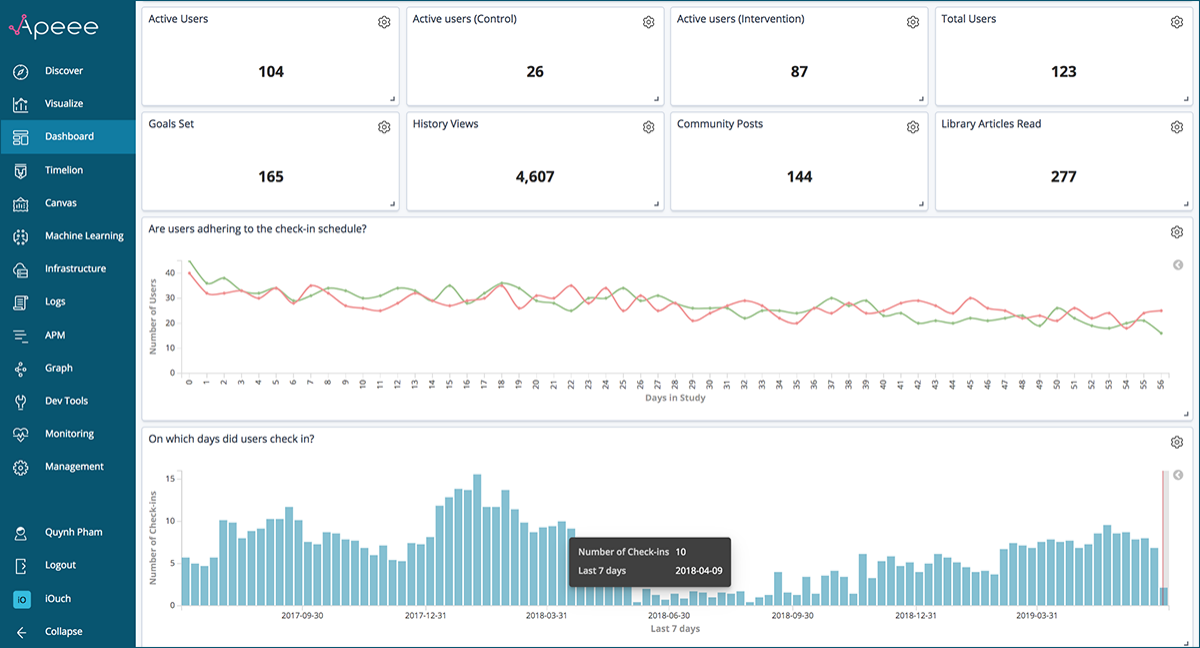
Screenshot of the Analytics Platform to Evaluate Effective Engagement research coordinator dashboard.

One key attribute of APEEE that eased its acceptance and adoption by research staff was the degree to which the platform could be customized according to staff roles. As part of the service of delivering APEEE, we embedded a needs assessment into our prestudy interview with staff. We were consequently able to build out custom dashboards and visualizations with these needs in mind. Figure 1 presents an APEEE dashboard designed for a research assistant whose primary task is to make weekly calls to study participants and encourage them to engage with *iCanCope*. This dashboard has focused content: only 4 analytic indicators are displayed, and the sidebar has minimal features. Figure 2 presents an APEEE dashboard for a research coordinator who manages multiple streams of research activity, from monitoring participant recruitment and adherence to the study protocol to conducting statistical analyses across study outcomes. In contrast, this dashboard covers a breadth of analytic indicators (only 10 are visible but 12 additional indicators can be viewed through scrolling down the dashboard), allows access to more features, and also has data export functionality to support offline work and interoperability with other analytic resources. Staff responded positively to the adaptable nature of the platform and took advantage of this capacity for customization through making numerous requests to “tweak” (P9) existing dashboards or build new ones to support different research tasks.

Although these requests were initially perceived by our research team as a sign of implementation success, it was only after fulfilling them that we noticed iOUCH research staff were asking for visualizations that they did not actually use. During our on-site observation sessions, staff almost always accessed the same two analytic indicators: (1) a data table of participants who had checked into *iCanCope* alongside the number of check-ins they had completed and the date of completion, and (2) a metric of the number of active users, defined as users who had generated any log data. This behavior was highlighted toward the end of the field study when our internal server unexpectedly reached a log data storage limit, forcing our research team to create *lite* versions of all APEEE dashboards with a subset of analytic indicators to maintain performance. When asked in their poststudy interview whether only having access to a limited number of indicators had affected the utility of the platform, staff commented that they “hadn’t even noticed a difference” (P2). The majority of indicators that had been made available to them were “interesting” (P13) and “nice to have” (P1), but ultimately did not change or improve their daily practices. However, they elaborated that had APEEE been taken down completely, this would have significantly impeded the data extraction and analysis routines that had been established around the platform.

Several characteristics of the iOUCH lab and its evaluative practice may have served as organizational antecedents for research innovation. APEEE’s introduction into the lab was championed by senior investigators who directed the research agenda and had good managerial relations with research staff. They were able to convince staff that the platform—and research analytics more broadly—was synergistic with existing projects and would facilitate research processes. In terms of staff competence, we noted a high *absorptive capacity for new knowledge*, defined by Greenhalgh et al as “a combination of formal expertise, informal organizational know-how, technical infrastructure, and relevant interpersonal networks” [28]. Finally, there was sufficient *organizational slack* within the lab, defined by Greenhalgh et al as “spare time, money, or expertise that can be channeled into new projects” [28], for staff to bear the onboarding process and develop confidence in using APEEE.

Unfortunately, this organizational slack did not extend to *methodological slack*, which we define as the capacity to pursue study designs and methods that significantly deviate from current research practice. Despite high interest and intention for APEEE to change evaluative practice, the iOUCH lab was ultimately bound by the commitments they had made to evidence their apps using RCTs. We observed the group discovering through APEEE that users were not adhering to the study protocol (ie, not using the app to report their symptoms once a day, every day, for 56 days) and also failing to use certain app features and functionality. However, staff had no bandwidth to react to these analytic insights, given the methodological rigidity of the RCT design to maintain internal validity [52]. This tension played out in surprising ways, the most notable being that staff eventually realized the limitations of applying APEEE to effect methodological change within an RCT-locked environment, yet still spoke highly of future opportunities beyond the field study to conduct data-driven trials. It is worth noting that toward the end of the field study, the lab had started drafting a secondary analysis of their RCT data comprising subgroups identified through APEEE and more broadly exploring alternative research designs (eg, sequential multiple assignment randomized trials and real-world observational studies) to maximize platform utility [53].

A critical factor that both enabled and constrained APEEE’s capacity to effect methodological change in the iOUCH lab was the underlying *iCanCope* data model. Before APEEE’s connection to *iCanCope*, there was no working hypothesis or data analysis plan to characterize the relationship between engagement and health outcomes. As a result, the set of analytic tags built out to log events and generate data for ingestion into APEEE did not capture the full breadth and depth of interactions that could have denoted effective engagement with the app, defined as “sufficient engagement to achieve intended outcomes” [54]. Furthermore, the structure and nomenclature of the tags themselves made it difficult to visualize the desired analytic indicators on APEEE, given the specific requirements of the platform. This architectural incompatibility allowed us to grasp the interdependencies between the data going into APEEE and the insights coming out. From this, we identified the need to expand the service of APEEE to include consultations with groups on (1) the research questions they want the platform to help them answer, (2) the parameters of their current data model and analytic tags to answer select questions, and (3) the amendments or additions to their data infrastructure required to answer all questions—to be done by their development team or our own.

Our findings suggest that the capacity for research analytics to optimize digital health evaluations is highest when there is (1) a collaborative working relationship between research client and analytics service provider, (2) a data-driven research agenda, (3) a robust data infrastructure with clear documentation of analytic tags, (4) in-house software development expertise, and (5) a collective tolerance for methodological change. Although these success factors are not listed in any particular order, we wish to emphasize the significance of establishing trust when introducing research analytics into evaluative practice. When asked to specify facilitators of APEEE’s introduction into the iOUCH lab, research staff unanimously cited the support provided by our research team as a determinant of success. They espoused the *process* of implementing APEEE—meeting to review the *iCanCope* data model and select appropriate analytic indicators, codesigning and iterating on dashboard design and content, engaging in meaningful dialog about analytic insights—as being more valuable than the *product* itself. This response supports our intent to position the platform as the technical *hard core* of a proposed research analytics service, with a suite of adaptable evaluation services forming the *soft periphery* [55,56]. Multimedia Appendix 1 presents our preliminary service blueprint of this research analytics service, which draws together the human and technical interactions and interdependencies required to implement this service in evaluative practice.

### Microlevel Findings

A total of 21 digital health innovators agreed to be interviewed for this research; reasons for denying the request included lack of availability (n=9) and professed lack of knowledge on analytics and evaluation (n=3). On average, innovators were young, male, educated, and worked in academia on mobile-based digital health interventions. Table 2 details their demographic characteristics, and Multimedia Appendix 2 presents the semistructured interview script.

In all the interviews we conducted, regardless of sector, role, or rank, innovators were able to clearly communicate the problem or unmet need that their product aimed to address. They provided empathetic and nuanced descriptions of how chronic diseases “totally change the way people go about their everyday lives” (P7) and were able to explain complex clinical concepts (eg, prostate-specific antigen nadir, and etiology of heart failure) without formal medical training. Innovators were “excited” (P10), “proud” (P12), and “satisfied” (P17) to work on projects that “made a real difference” (P6) and were “on the cutting edge of health care” (P8). When asked to describe how their product aimed to solve the problem they had identified, most innovators referenced a conceptual or logic model that delineated the solution. These innovators also provided measured estimates of their product’s potential effect on health outcomes, often adding caveats that more “research” (P19), “users” (P16), or “time” (P3) was required to validate claims. In contrast, a small number of innovators found it difficult to convey the mechanisms of change that their product would facilitate to improve health outcomes. They also held ambitious beliefs of their product’s capacity to positively impact health and well-being. When probed to substantiate the rationale supporting these beliefs, innovators were hesitant to elaborate and defaulted to reiterating product features and functionality.

Overall, innovators were able to communicate the ideal or intended user journey for their products with ease and were confident in their assessment of what constituted effective engagement with their product. All innovators defined effective engagement quantitatively (ie, amount, duration, breadth, or depth of intervention usage); some identified a single event that was critical to users deriving any benefit, whereas others listed a series of sequential events. We noted a shared prioritization on events that involved capturing data, for example, entering blood glucose readings or logging asthma exacerbations. When asked whether users were effectively engaging with products as intended, half of all innovators disclosed that they were “not sure” or “needed to check.” The other half said yes; however, only 5 innovators referenced formative research or definitive trials to support their claims.

**Table 2:** Demographic characteristics of digital health innovators.

Characteristics			Values
Age (years), range			22-45
Age (years), mean (SD)			30.4 (5.3)
Age (years), median (IQR)			29 (4)
**Gender, n (%)**			
	Male	13 (62)	
	Female	8 (38)	
**Education, n (%)**			
	Graduate	17 (81)	
	Undergraduate	4 (19)	
**Sector, n (%)**			
	Academic	18 (86)	
	Industry	3 (14)	
**Occupation, n (%)**			
	Research coordinator	4 (19)	
	Research analyst	3 (14)	
	Designer	3 (14)	
	Developer	3 (14)	
	Manager	3 (14)	
	Director	2 (10)	
	Product owner	2 (10)	
	Research associate	1 (5)	
**Innovation platform, n (%)**			
	Mobile	13 (62)	
	Web	8 (38)	
**Clinical focus of innovation, n (%)**			
	Well-being	4 (19)	
	Heart failure	4 (19)	
	Chronic pain	3 (14)	
	Juvenile idiopathic arthritis	2 (10)	
	Mental health	2 (1)	
	Prostate cancer	2 (10)	
	Sickle cell disease	2 (10)	
	Asthma	1 (5)	
	Diabetes	1 (5)	

Of the 21 innovators interviewed for this research, every single one included the word *data* in their definition of analytics. Innovators broadly defined the practice of analytics as the collection, aggregation, or visualization of data to generate actionable insights. They sought to differentiate “raw data” (P11) from analytics, which they saw as “data with meaning” (P7) that could be used to “understand the current state of a product and predict how people might use it in the future” (P15). They also endorsed the notion that “data never lies” (P5) and consequently perceived analytics to be a valid source of information to “help with big decisions” (P17). Innovators intuitively considered analytics to be a quality improvement or project management initiative and did not mention its application in evaluative practice. They valued the opportunity provided by analytics to “sanity check design and development assumptions” (P18) and “get to know users better” (P4), with a final aim of translating this knowledge into product specifications. They also emphasized an expectation that analytic insights be generated in real time and visualized in a way “that didn’t take much time to read through and understand” (P11). Almost all innovators referenced the Google Analytics platform when describing their familiarity with analytics in practice; some even initiated their definition of analytics with mention of the platform. Interestingly, of those 19 innovators, only 4 had ever personally used the platform and found it to have a “steep learning curve” (P4), “really confusing to use” (P10), and “totally impossible to segment users into useful subgroups for analysis” (P21).

Despite these challenging experiences with Google Analytics and an overall lack of experience with analytics across the group, innovators unanimously expressed interest in applying research analytics to evidence their products. They also shared similar preferences for how analytic insights should be presented, namely through a dashboard interface with dynamic widgets containing aggregate-level analytic indicators. However, there were striking sectoral differences in the quality of evidence innovators believed research analytics could generate, as well as its intended use. Industry innovators espoused the opportunity for analytics-enabled research to “replace trials” (P20) and “generate real-world evidence” (P18) to support procurement efforts and improve market valuation. They disclosed that they were already collecting usage logs and patient-reported outcomes but lacked a resource to collate these data and interpret insights. In contrast, academic innovators saw value in conducting research analytics to inform subgroup analyses for a definitive trial or to generate quantitative data to complement qualitative research. As a stand-alone source of evidence, there was consensus among academic innovators that analytic insights placed “near the bottom of the evidence pyramid” (P2) and were insufficient on their own to demonstrate efficacy [57]. However, academic innovators saw value in research analytics as a part of a broader “data-driven research agenda” (P1), and affirmed that applying analytic insights to optimize digital health interventions might help them to “survive definitive trials and demonstrate efficacy” (P9). Other areas of application proposed by the group included (1) monitoring the status of multisite studies to ensure standardized study protocol execution; (2) conducting A/B testing on product features and functionality to “test out behavioural hunches” (P15); (3) identifying “dying or dead” (P19) intervention components that had limited effects on outcomes; and (4) informing strategies to promote adherence and prevent disengagement from both the study and the product.

Although innovators were encouraged by the proposed benefits of research analytics, they also acknowledged numerous barriers to using analytic insights in practice. Executive-level innovators divulged that they often made decisions based on “intuition” (P21) or “gut feeling” (P15), and were unsure of how they would react to data that conflicted with their convictions. All three developers raised issues of personal health information being openly used in a nonclinical context and cautioned that consent forms and terms of agreement would need to be updated to reflect this “off-label use of log data” (P5). Across the group, the most cited challenge to adopting research analytics was a collective reservation that analytic insights could be “trusted.” Some innovators voiced concerns that users often behaved in “random and unpredictable ways” (P10), and thus, the data they generated might not be reflective of their self-care intentions. Others indicated that they would need to be certain of log data quality, specifically “data accuracy and comprehensiveness” (P9), before using analytic insights to inform decision making.

## Discussion

### Principal Findings

This study illustrates the complexity of implementing research analytics in evaluative practice to evidence a digital health intervention, taking into account the political, organizational, and personal contexts that influence methodological change. Our findings confirm that the practice of research analytics is an efficient and effective means of supporting digital health evidence generation. The introduction of an APEEE with digital health interventions into a busy research lab was ultimately accepted by the research staff, routinized in their evaluative practice, and optimized their existing mechanisms of log data analysis and interpretation. By the end of the 6-month field study, the research group had arranged to sustain this innovation past the field study period to support future digital health evaluations. Although these findings suggest that research analytics may be integral to modernizing digital health research models, the process of effecting methodological change was not trivial. Despite emerging policy and regulatory interest in digital health evaluation and a primed research organization with engaged staff, establishing a new model of digital health evaluation was challenging. The difficulties of changing evaluative practice to accommodate analytic methodologies were largely attributable to (1) a discrepancy between perceived analytic wants and actual analytic needs when conducting digital health evaluations, (2) a nascent data infrastructure that was not architected to generate robust analytic insights, and (3) a lack of methodological slack to trial data-driven study designs and methods.

The emergence of methodological slack as a distinct system antecedent for digital health research innovation is significant in its recognition of the tangible constraints imposed by positivist research paradigms and traditional models of scientific inquiry [58]. Although digital health innovations are increasingly recognized as complex interventions that deliver care within complex health systems [59], conventional approaches to evidencing them remain wedded to definitive trials that assume linear causality and control for complexity [60]. This theoretical orientation comes at a cost, namely, the incapacity to adopt data-driven research designs and methods that are dynamic in process and assume emergent causality. We posit that a lack of methodological slack is similar to the phenomenon of *paradigm paralysis*, which is defined as “the inability or refusal to shift worldviews and see beyond current theoretical models of thinking” and acknowledged as a “block to creativity, innovation, and change” [61]. From our exploration of macro- and microlevel contexts that influence digital health evidence generation, we offer two factors that may be perpetuating this positivist bias: (1) the role of federal funding agencies in directing the provincial digital health research agenda and (2) the assessment of log data and analytic insights as a weaker form of scientific evidence.

Although there are sparse pockets of provincial funding earmarked for digital health evidence generation, the majority of this research is federally funded through the Canadian Institutes of Health Research (CIHR). As the leading federal agency responsible for funding health and medical research in Canada, CIHR directs the provincial digital health research agenda through releasing strategic funding calls that target key areas of research. Funding calls are often prescriptive on methodological approach, for example, a recent call for “applications focused on the development, integration and evaluation of electronic health innovations…to optimize the outcomes of patients experiencing transitions in care” required the use of a “pragmatic randomized clinical trial methodology” and provided a list of suggested trial designs [62]. These directives illustrate a significant criticism of CIHR: that it is “positivist in orientation” and designed to fund “short-term, hypothesis-driven evaluative studies” [63]. As a consequence, research groups such as iOUCH are incentivized to conduct definitive trials, even if such trials may not suit the immaturity of the intervention and are less likely to demonstrate success.

In recent years, CIHR has developed its mandate to be more inclusive of alternative research paradigms. The agency acknowledges that “publicly-funded research is problem-driven, and the nature of science itself has changed with the move from positivist linear explanations to complex systems-based research and explanatory models” [64]. To operationalize this mandate and “change the paradigm of how research is rewarded,” CIHR launched the Innovative Clinical Trials (iCT) Initiative in 2016 with an annual budget of Can $11.7 million to “support the development and adoption of innovative and cost-effective trial methodologies” that are “alternative to traditional RCTs” and aim to *“*reduce the cost of conducting trials, reduce the amount of time needed to answer research questions, increase the relevance of research findings to patients, health care providers and policy makers…and maximize the use of existing knowledge and data” [65]. Examples of iCT designs provided by CIHR include adaptive, n-of-1, registry, and observational trials [65]. A total of 3 years on from the first iCT funding call, evidence of the initiative’s impact on shifting research programs to include innovative research methodologies was demonstrated during our research timeframe: in March 2019, the iOUCH lab was awarded an iCT Catalyst Grant to conduct a multiple baseline study of *iCanCope*, which will include the use of APEEE to monitor weekly outcomes data and establish stable baselines [53]. Moreover, of the 20 catalyst grants that have been awarded since 2016, 4 have funded innovative trials of digital health interventions and 2 have specifically funded data-driven optimization trials, which we contend are the most ideal study designs to support through research analytics [66]. This progress suggests that strategic federal funding initiatives such as iCT are an effective policy lever for promoting acceptance and application of analytics-enabled research to optimize digital health evidence generation.

Our interviews with digital health innovators revealed a reluctance to adopt research analytics as a method of evidence generation owing to a lack of trust in the log data quality powering analytic insights. This unease was more pronounced in academic innovators than their industry counterparts and manifested itself in the limited degree to which this sector was willing to consider analytic insights as a viable outcome measure to demonstrate efficacy. This finding builds on a burgeoning body of literature highlighting the use of flawed, uncertain, proximate, and sparse (FUPS) data to inform research and care [67]. From our findings, we posit that log data generated from users engaging with digital health interventions fit the criteria for FUPS data. Engagement log data may be flawed, due to missing data or erroneously logged events; uncertain, due to differences in how data are behaviorally conceptualized along an engagement continuum; proximate, in that analytic indicators of engagement indicate that users may be engaging effectively with a digital health intervention but do not definitively confirm a relationship between engagement and intended outcomes; and sparse, in that a low volume of events within key subgroups due to disengagement and attrition may limit the possibility of statistical inference. In their case study of a large-scale FUPS dataset of child mental health outcomes following contact with specialist mental health services in the United Kingdom, Wolpert and Rutter opine that clinical researchers are heavily influenced by the paradigm of evidence-based medicine and trained to interrogate data by its ranking on the evidence hierarchy [68]. As a result, they may be predisposed to criticize or dismiss FUPS data, particularly if such data challenge strongly held convictions or interests. The authors further note that FUPS data are often used in charged and contested contexts where conclusions drawn from them have significant implications; careful thought must be given to how these data are weighted for decision making.

In the context of digital health evidence generation, we acknowledge that the practice of research analytics may be compromised by the FUPS nature of digital health log data. However, we also acknowledge the harsh realities of the current digital health research landscape, namely the paucity of evidence-based digital health interventions currently available to consumers and the “data-poor” approaches to evidencing them that cannot keep pace with technologic change [4]. At a time of unsustainable growth in the burden and cost of chronic disease management [69], the promise of digital health to transform care has not been delivered. We contend that a new standard of digital health evidence that is proportionate to the risk of the intervention and the magnitude of the decision being made will allow for the consideration of log data as a valid data source to optimize interventions and improve decisions. To safeguard against drawing misleading conclusions, we recommend that digital health innovators adhere to three key principles proposed by Wolpert and Rutter for analyzing FUPS data: (1) be honest and upfront about the limitations of log data to produce causal inferences, (2) be transparent in the statistical analyses used to derive analytic indicators, and (3) triangulate analytic insights with other information [68]. In doing so, these data can support meaningful dialogue between key stakeholders, including policy makers, regulators, innovators, and users, in relation to the impact of digital health innovations on health and care.

### Strengths and Limitations

Through this research, we were able to comprehensively study and document the implementation of research analytics in digital health evaluative practice. Working with front-line and senior research staff, we developed standard operating procedures, technical guidance documents, and research protocols for setting up and running a research analytics practice. We collected rich qualitative data across macro, meso, and micro contexts to characterize the complexity of designing and implementing the service of research analytics, and identified the barriers and facilitators to scale, spread, and sustain this service in Ontario. We were able to operationalize this service and gain detailed insights into organizational routines and how these changed with the provision of APEEE. A trial phase of APEEE continues within the iOUCH lab, and further work is ongoing to extend the service to other research groups and evaluation settings.

The findings of our research must be viewed in light of their limitations. First, we had difficulty recruiting innovators from industry into this study owing to their demanding schedules and a lack of obvious return on investment; as such, industry narratives may be underrepresented. We engaged in nonprobability purposive sampling to recruit digital health innovators for interviews, which may have biased our sample selection and limited the generalizability of research findings. To mitigate this bias, we expanded our sampling frame to include innovators who could be interviewed virtually or on-site at international academic conferences. Second, our research focused on the implementation of research analytics in a single digital health research lab. Although we assert that the iOUCH lab is representative of a typical evaluation setting, it is possible that a different lab with no prior relation to our research group may have found the implementation of APEEE more difficult and less beneficial. Finally, the capacity to generalize findings from this research was a trade-off that we considered carefully when selecting the case study methodology to direct this research. Unlike quantitative study designs, the goal of case studies is to produce *analytic generalizations*, defined by Yin as “the degree to which findings bear upon a particular theory, theoretical construct or theoretical sequence of events” [70]. Analytic generalizations are distinct from statistical generalizations in that they do not draw inferences from data to a population. Instead, analytic generalizations compare the results of a case study with a previously developed theory and seek to generalize theoretical insights as opposed to actual study results. In our case study, the emergence of methodological slack was a form of analytical generalization that drew from Greenhalgh et al’s theoretical construct of organizational slack [27] and may be applied to future cases of implementing research analytics in digital health evaluative practice. The majority of insights from our three tiers of analysis (eg, our suggestions on the capacity for research analytics to optimize digital health evaluations) can and should be characterized as analytic generalizations.

### Conclusions

Scientific methods and practices that can facilitate the agile trials needed to iterate and improve digital health interventions warrant continued implementation. As outlined in this paper, the service of research analytics may help to accelerate the pace of digital health evidence generation and build a data-rich research infrastructure that enables continuous learning and evaluation. Valid concerns exist regarding the formation of unfounded or opportunistic causal inferences based on flawed analytic insights. However, continuing to pursue evaluative practices that fail to raise the standard of digital health safety and effectiveness is untenable. Our research offers compelling reasons to continue exploring the potential of research analytics to advance innovative methodologies and optimize the quality and impact of digital health care.

## Appendix

Multimedia Appendix 1 Analytics Platform to Evaluate Effective Engagement (APEEE) service blueprint.

Multimedia Appendix 2 Micro level semistructured interview script.

## Abbreviations

APEEE: Analytics Platform to Evaluate Effective Engagement
CIHR: Canadian Institutes of Health Research
COE: Center of Excellence
EPR: electronic patient records
FUPS: flawed, uncertain, proximate, and sparse
iCT: Innovative Clinical Trials
iOUCH: Improving Outcomes in Child Health through Technology
MOHLTC: Ministry of Health and Long-Term Care
RCT: randomized controlled trial
