# Supplementary figures and images for: The Service of Research Analytics to Optimize Digital Health Evidence Generation: Multilevel Case Study
Source: J Med Internet Res. 2019 Nov 11;21(11):e14849. doi: 10.2196/14849 (PMC6878108; doi:10.2196/14849)

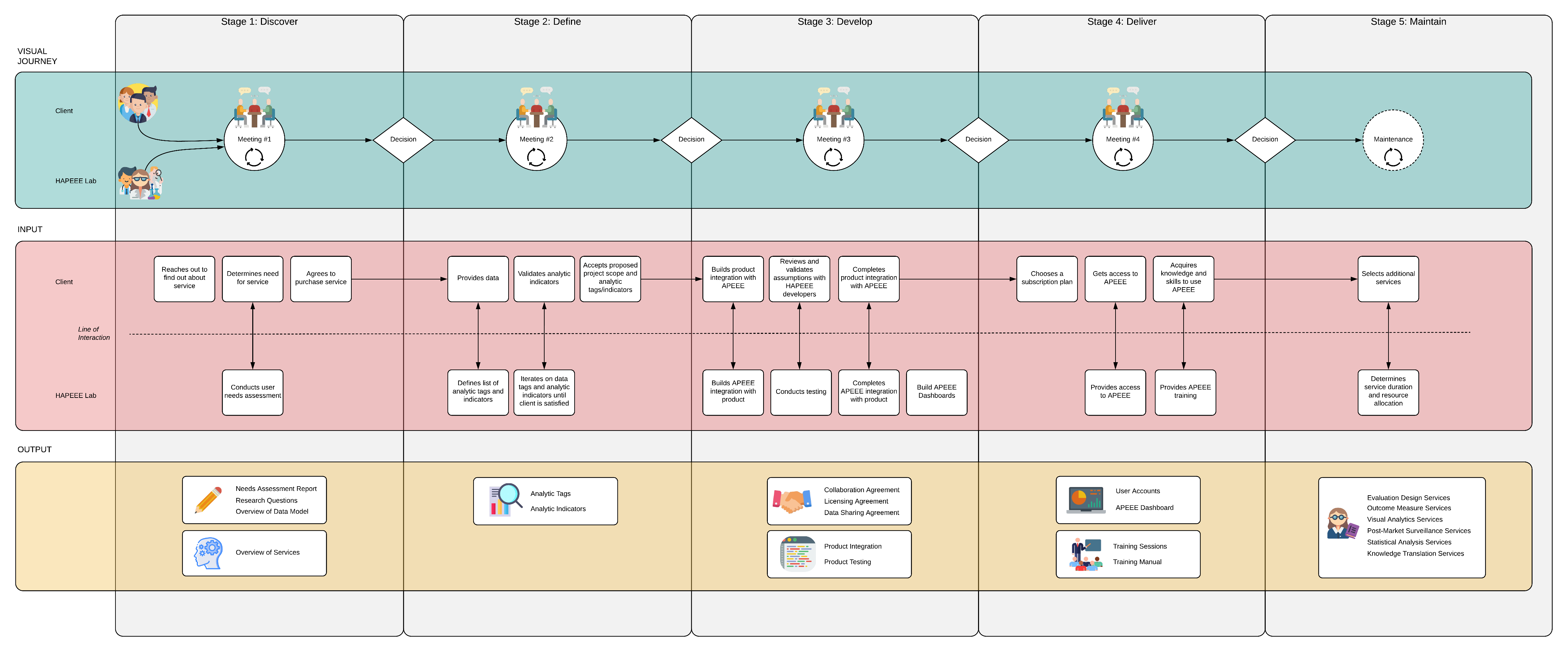

Supplement: Multimedia Appendix 1 [file jmir_v21i11e14849_app1.png]
