# Supplementary material for: The Service of Research Analytics to Optimize Digital Health Evidence Generation: Multilevel Case Study
Source: J Med Internet Res. 2019 Nov 11;21(11):e14849. doi: 10.2196/14849 (PMC6878108; doi:10.2196/14849)
Supplement: Multimedia Appendix 2 [file jmir_v21i11e14849_app2.pdf]

Multimedia Appendix 1. Micro level semi-structured interview script

1. Please tell me a little about yourself, your current role, and a bit of background history on how you ended up in this role.

2. I would like to know more about the digital health product that you are currently working on. Would you be able to first describe to me the problem or unmet need that the product aims to address?

3. Can you please describe to me how the product aims to address this problem or unmet need?

4. What do you believe is the impact the product will have on the health and wellbeing of its users?

5. Can you walk me through the ideal or intended user journey for the product?

6. What do you consider to be ‘effective engagement’ with the product?

7. What is your understanding of the word ‘analytics’?

8. Do you currently use analytics as part of your work?

9. Based on your understanding of the word ‘analytics’, what do you think an analytics platform for looking at digital health log data might look like?
   - What features and functionality do you think it should have?

10. Would you use an analytics platform for looking at digital health log data in your own work? Why or why not?

11. In the context of your own work, how do you see such a resource helping you?

12. What barriers do you foresee for using it?

13. How might it change your day-to-day work?
